# 503. Burn Camp Participation Is Associated with Higher Work-Related Quality of Life in Firefighters

**DOI:** 10.1093/jbcr/irag033.162

**Published:** 2026-04-06

**Authors:** Micayla Kotarski, Emily Beutler, Chandler Kingsbury, Robert Marriott, Callie M Thompson

**Affiliations:** University of Utah Health Burn Center, Salt Lake City, UT; University of Utah, Salt Lake City, UT; University of Utah Health Burn Center, Salt Lake City, UT; University of Utah Health Burn Center, Salt Lake City, UT; University of Utah Health, Salt Lake City, UT

## Abstract

**Introduction:**

Firefighters (FFs) are the foundation of community safety in the United States. These first responders play a critical role in the care of the burn patients. The nature of the work requires these individuals to be exposed to repetitive secondary trauma in high-stress and high-risk environments. Ensuring the long-term comprehensive health of the FF workforce will be critical in the continued successful delivery of burn care. We sought to understand the Work-Related Quality of Life (WRQoL) in local FFs to develop future interventions to improve WRQoL and resilience. One intervention we evaluated was their participation in our local Burn Camp (BC) programs.

**Methods:**

After IRB approval, we sent quarterly anonymous surveys via email to members of our local firefighter union between August 2024 and June 2025. We collected minimal demographic data and inquired about WRQoL & BC participation. Standard statistical analyses were performed in Stata18.

**Results:**

Demographics were as follows: 57% FF, 30% Paramedic, and 12% Management, 1% EMTs. Most (81%) had >6 years on the job. Ten% participated in BC. Responses to the question of satisfaction with the overall quality of working life by quarter are presented in Table 1, there were no significant differences across time points. BC participation was associated with satisfaction with working life (100%, *p*=.023) and less thoughts of leaving the job (0% had thoughts of leaving every few months or more, *p*=.023).

**Conclusions:**

This study highlights the generally stable WRQoL among our local firefighters over the course of the year 2024-2025. However, participation in BC programs was significantly associated with greater satisfaction in overall working life and fewer thoughts about leaving the profession. These findings suggest that structured, meaningful engagement outside of traditional emergency response roles, such as Burn Camp, may positively impact firefighter well-being and retention. A larger study should be done to evaluate if this trend holds beyond a single regional community.

**Applicability of Research to Practice:**

Future interventions aimed at improving resilience and job satisfaction in this workforce should consider incorporating similar community-based, purpose-driven opportunities.

**Funding for the study:**

N/A.

